# Proteomics identifies lipocalin-2 in neonatal inflammation associated with cerebrovascular alteration in mice and preterm infants

**DOI:** 10.1016/j.isci.2023.107217

**Published:** 2023-06-27

**Authors:** Giacomo Gravina, Maryam Ardalan, Tetyana Chumak, Anders K. Nilsson, Joakim C. Ek, Hanna Danielsson, Pernilla Svedin, Milos Pekny, Marcela Pekna, Karin Sävman, Ann Hellström, Carina Mallard

**Affiliations:** 1 Institute of Neuroscience and Physiology, Sahlgrenska Academy, University of Gothenburg, Gothenburg, Sweden; 2 Translational Neuropsychiatric Unit, Department of Clinical Medicine, Aarhus University, Aarhus, Denmark; 3 Section for Ophthalmology, Department of Clinical Neuroscience, Institute of Neuroscience and Physiology, Sahlgrenska Academy, University of Gothenburg, Gothenburg, Sweden; 4 Centre for Translational Microbiome Research, Department of Microbiology, Tumor and Cell Biology, Karolinska Institutet, Stockholm, Sweden; 5 Sach’s Children’s and Youth Hospital, Södersjukhuset, Stockholm, Sweden; 6 Laboratory of Astrocyte Biology and CNS Regeneration, Center for Brain Repair, Department of Clinical Neuroscience, Institute of Neuroscience and Physiology, Sahlgrenska Academy at the University of Gothenburg, Gothenburg, Sweden; 7 University of Newcastle, Newcastle, NSW, Australia; 8 Florey Institute of Neuroscience and Mental Health, Parkville, VIC, Australia; 9 Laboratory of Regenerative Neurobiology, Center for Brain Repair, Department of Clinical Neuroscience, Institute of Neuroscience and Physiology, Sahlgrenska Academy at the University of Gothenburg, Gothenburg, Sweden; 10 Department of Pediatrics, Institute of Clinical Sciences, University of Gothenburg, Sahlgrenska Academy, Gothenburg, Sweden; 11 Region Västra Götaland, Department of Neonatology, The Queen Silvia Children’s Hospital, Sahlgrenska University Hospital, Gothenburg, Sweden

**Keywords:** Neurology, Immunology, Proteomics

## Abstract

*Staphylococcus (S.) epidermidis* is the most common nosocomial coagulase-negative staphylococci infection in preterm infants. Clinical signs of infection are often unspecific and novel markers to complement diagnosis are needed. We investigated proteomic alterations in mouse brain after *S. epidermidis* infection and in preterm infant blood. We identified lipocalin-2 (LCN2) as a crucial protein associated with cerebrovascular changes and astrocyte reactivity in mice. We further proved that LCN2 protein expression was associated with endothelial cells but not astrocyte reactivity. By combining network analysis and differential expression approaches, we identified LCN2 linked to blood C-reactive protein levels in preterm infants born <28 weeks of gestation. Blood LCN2 levels were associated with similar alterations of cytokines and chemokines in both infected mice and human preterm infants with increased levels of C-reactive protein. This experimental and clinical study suggests that LCN2 may be a marker of preterm infection/inflammation associated with cerebrovascular changes and neuroinflammation.

## Highlights

• *Staphylococcus epidermidis* induces proteomic alteration in the immature mouse brain
• Lipocalin-2 is associated with cerebrovascular changes and astrocyte reactivity
• Blood Lipocalin-2 is linked to blood C-reactive protein levels in preterm infants
• Lipocalin 2 might be an additional marker of infection in preterm infants

## Graphical abstract

**Figure:**
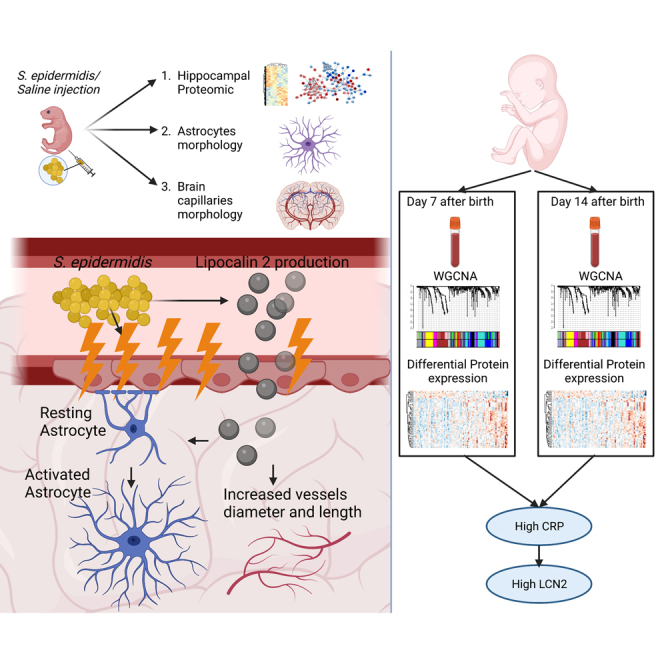

## Introduction

*Staphylococcus (S.) epidermidis* is one of the most common bacteria that colonizes skin and mucous in mammals, including humans.^1^ *S. epidermidis* has long been considered an innocuous and opportunistic bacterium. However, the commensal or beneficial role of *S. epidermidis* in healthy hosts has recently been questioned, especially in preterm infants.^2^ Bloodstream infections account for 11–19% of neonatal mortality worldwide.^3^ Owing to prolonged hospitalization and invasive treatments, preterm infants have a higher risk of biofilm-related infections, including *S. epidermidis*.^4^ Infection in the perinatal period has detrimental effects on brain development^5^ and is associated with a higher risk of developing neurodevelopmental disorders.^6^^,^^7^^,^^8^^,^^9^ Several studies have addressed *S. epidermidis*-related pathology as an important contributor to neonatal morbidity in preterm infants.^2^ Clinically, postnatal sepsis, predominantly coagulase negative staphylococci, in preterm infants is implicated in white matter abnormalities and neurodevelopmental impairment.^10^^,^^11^ However, only a few studies have focused on the effects of *S. epidermidis* on neurodevelopment. We previously showed that *S. epidermidis* infection reduced gray and white matter volume in neonatal mice^12^ and sensitized the neonatal brain to hypoxic-ischemic (HI) injury.^13^^,^^14^ Recently, we demonstrated that *S. epidermidis* induces activation of microglial cells in synergy with increased blood-brain barrier (BBB) permeability and peripheral cell infiltration.^15^

Owing to the lack of pyrogenic events and/or the reduced arsenal of toxins released, the clinical signs of infection, including *S. epidermidis*, can be subacute or chronic and nonspecific, making the diagnosis difficult.^16^ Thus, additional markers of infection are needed to complement the diagnostic toolbox for infection in preterm infants. Currently, the diagnosis is based on the measurement of serum C-reactive protein (CRP) levels, which alone is unlikely to aid in early diagnosis of late-onset infection.^17^ For this purpose, we aimed to explore the impact of infection in the neonatal mouse brain and its associated pathways. Although, the primary aim of the current study was to identify molecular changes associated with infection/inflammation in both mice and human, the concomitant increase in LCN2, CRP and IL6, that we found in this study, might aid in recognizing preterm infants experiencing infection.

Proteomic analysis has the advantage of examining complex biological functions involving large numbers and networks of proteins and has been shown to be a useful tool to identify markers of disease progression in several neurological conditions in the adult as well as finding biomarkers that can predict the risk of a particular disease.^18^ In the present experimental and clinical study, by employing proteomic analysis in both neonatal mice and preterm infants, we identified LCN2 as a promising marker for preterm infection/inflammation associated with cerebrovascular alterations and neuroinflammation.

## Results

### *S. epidermidis* infection changes the proteome in the immature hippocampus

**Figure 1:**
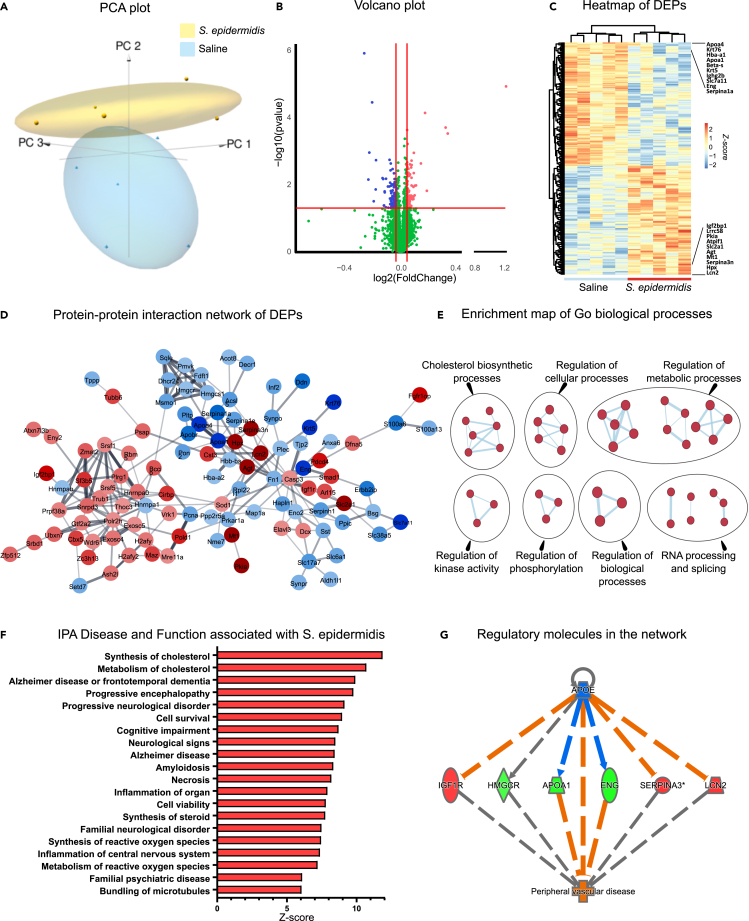
Changes in hippocampal protein expression following *S. epidermidis* infection (A) Principal component analysis of hippocampal proteomics shows a clear separation of samples from animals treated with saline and *S. epidermidis* at 24 h post-infection (saline n = 5, *S. epidermidis* n = 5). (B) Volcano plot showing the spread of 5914 differentially expressed proteins (71 downregulated, blue and 78 upregulated, red, see also Table S1). (C) Heatmap of differentially expressed proteins. The top 10 down- and up-regulated proteins are enlarged and listed in the heatmap. (D) Protein-protein interaction network constructed from the 149 DEPs. The network was created using Cytoscape software. Blue and red indicate respectively the down- and up regulated proteins. (E) Enrichment map of significant gene ontology (GO) terms between *S. epidermidis* and control mice. Nodes represent gene sets. Highly similar protein sets are connected by edges, grouped in sub-clusters, and annotated automatically. (F) Top 20 significantly enriched IPA Diseases and functions (p < 0.01) sorted by *Z* score (red indicates positive *Z* score). (G) Upstream regulator analysis showing the effects of *S. epidermidis* infection. Red indicates increased and green indicates decreased measurements of proteins. Orange dashed lines predict activation, blue dashed lines predict inhibition and gray dashed lines indicate a non-predicted response (see also Table S2).

To gain insight into pathways perturbed 24h after *S. epidermidis* or saline injection, whole hippocampi of 5-day-old mouse pups were dissected and exposed to Tandem Mass Tag spectrometry to evaluate the protein profile. Principal component analysis showed clear separation in the proteomic profiles in *S. epidermidis*-infected and saline mice (Figure 1A). Out of a total of 5914, 149 proteins were differentially expressed between *S. epidermidis* and saline mice (p < 0.05 and FC >±1.1), among these 71 were downregulated and 78 were upregulated (Figure 1B and Table S1). Differentially expressed proteins (DEP) are illustrated in the heatmap with the top 10 down/up-regulated proteins indicated (Figure 1C). Of note, the most upregulated and downregulated proteins were LCN2 and ApoA4, respectively. To understand the functional interactions between DEPs, a protein-protein interaction (PPI) network was constructed. Based on the 149 DEPs, the PPI identified 147 nodes and 229 edges. These proteins were highly connected within the respective networks (Figure 1D). To gain further mechanistic insight, enrichment map analysis was performed.^19^ We found significant enrichment in multiple categories, including Cholesterol biosynthetic processes, Regulation of biological, cellular and metabolic processes, and RNA processes and splicing (Figure 1E).

To complement the results of the enrichment pathway analysis, we performed ingenuity pathway analysis (IPA) to identify functions, mechanisms and diseases associated with *S. epidermidis* infection. Similar to the enrichment analysis, cholesterol metabolism was predicted to be profoundly affected following infection (Figure 1F). We also identified mechanisms potentially involved in disease such as cell survival, necrosis, neuroinflammation and generation of reactive oxygen species. Of interest, many neurological diseases were associated with *S. epidermidis* infection such as Alzheimer's disease, progressive neurological disorder and cognitive impairment (Figure 1F), suggesting that these protein alterations might be involved in the development of neurological conditions. To further explore key proteins in the network, we used IPA to create a network consisting of regulatory molecules. This analysis aimed to find upstream regulators that can explain the protein changes in the overall network. We found that APOE, IGF1R, HMGCR, APOA1, ENG, SERPINA3 and LCN2 were hub proteins in the network (Figure 1G and Table S2), which were also predicted to be associated with peripheral vascular disease.

Jointly, these results indicate that in the hippocampus, *S. epidermidis* profoundly alters the expression of proteins which might be associated with neurological conditions and vascular impairments.

### LCN2 is upregulated in blood and hippocampus following *S. epidermidis* infection and co-localizes with brain vessels

**Figure 2:**
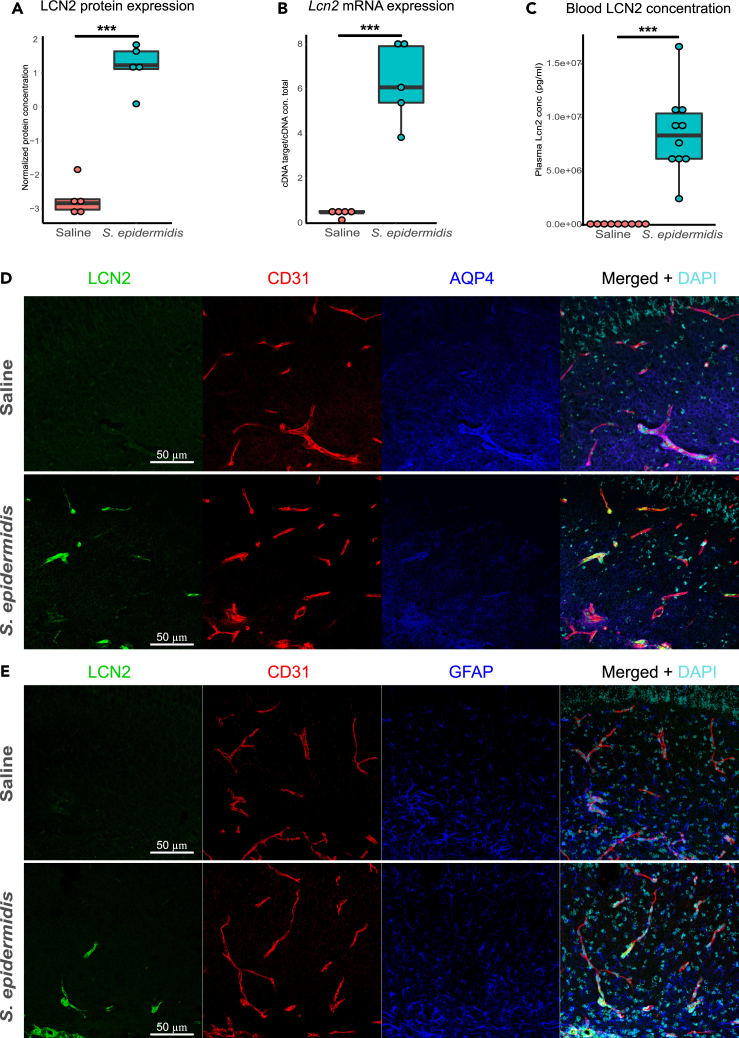
Upregulation of Lipocalin 2 following *S. epidermidis* and co-localization with blood vessels (A and B) Hippocampal protein and (B) mRNA expression of lcn2 24 h after induction of *S. epidermidis* infection (saline n = 5, *S. epidermidis* n = 5). (C) Blood Lcn2 protein concentration 24 h after *S. epidermidis* infection (saline n = 9, *S. epidermidis* n = 10). (D) Images of brain sections immunostained with anti-lcn2 (green), anti-CD31 (vessels-red), anti-AQP4 (astrocytes end-feet-blue) antibodies and DAPI (nuclei-turquoise) in the hippocampus of saline mice (top panel) and *S. epidermidis* infected mice (bottom panel). (E) Images of brain sections immunostained with anti-lcn2 (green), anti-CD31 (vessels-red), anti-GFAP (astrocytes-blue) antibodies and DAPI (nuclei-turquoise) in the hippocampus of saline mice (top panel) and *S. epidermidis* infected mice (bottom panel), see also Video S1. Data are presented as median, 10–90th percentile. Statistical comparison between the *S. epidermidis* and saline groups was performed using the non-parametric Mann-Whitney test. ∗∗∗p < 0.001.

To better understand the impact of *S. epidermidis* on the neonatal brain and the underlying molecular mechanisms, we focused on LCN2 as it was found to be the most upregulated protein in the network and one of the hub proteins identified by the IPA analysis. Proteomic analysis showed a 15-fold increase of the LCN2 level in hippocampus of *S. epidermidis*-infected mice (p < 0.0001, Figure 2A). Next, we tested the hypothesis that LCN2 is locally produced in the brain. We found that *Lcn*2 mRNA expression was dramatically increased in the hippocampus of *S. epidermidis*-infected mice compared to the saline group (p < 0.0001, Figure 2B) and was positively correlated with the relative LCN2 protein abundance (r = 0.93 and p = 0.0001). In blood samples collected from the same animals,^14^ LCN2 concentration was increased by *S. epidermidis* infection (Figure 2C). Further, to determine the cellular localization of LCN2 protein in the hippocampus of *S. epidermidis*-infected mice, immunofluorescence staining for LCN2 and Cluster of differentiation 31 (CD31), Aquaporin 4 (AQP4) or Glial fibrillary acidic protein (GFAP) on brain sections was performed. Consistent with the proteomic and gene expression results, LCN2 was detected in the hippocampus of *S. epidermidis*-infected mice (Figure 2D). We found that LCN2 mainly localized within CD31 positive endothelial cells but not in astrocyte endfeet (Figure 2D) or astrocyte soma (Figure 2E and Video S1), pointing to brain vessels as the major source of LCN2 induced by *S. epidermidis* infection.

Video S1. Co-localization of GFAP (blue), LCN2 (green) and CD31 (red), related to Figure 2

### *S. epidermidis* infection alters hippocampal vasculature

**Figure 3:**
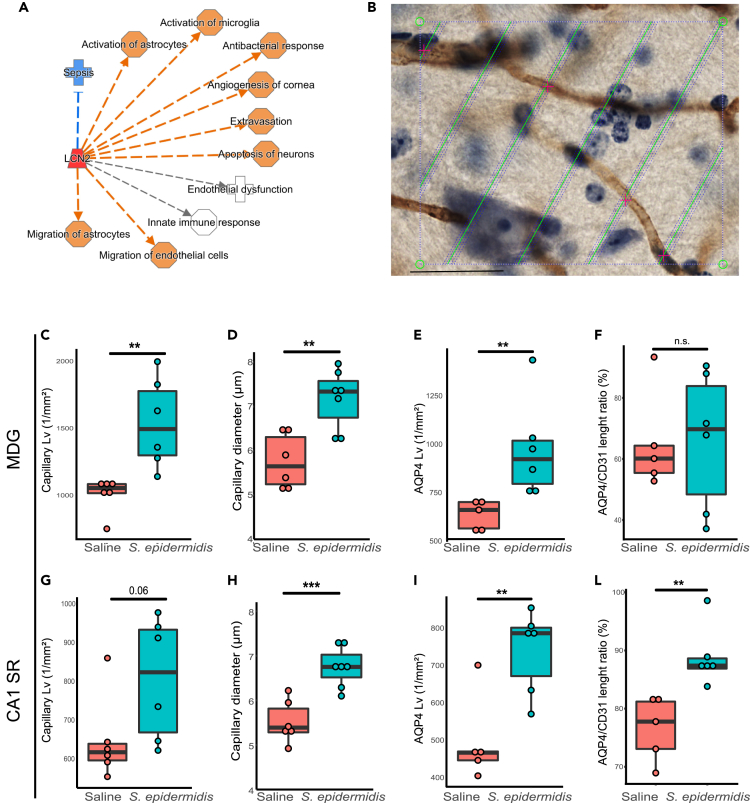
Cerebrovascular changes associated with *S. epidermidis* infection in the neonatal hippocampus (A) IPA regulatory network analysis identified an increase in lcn2 (red) and as a molecule involved in neuroinflammatory responses. Orange dashed lines predict activation, blue dashed lines predict inhibition and gray dashed lines indicate non-predicted responses. (B–I) Representative image of CD31^+^ immunostained capillaries (brown color). CD31 capillary length was quantified in PND5 mice (24 h after *S. epidermidis* infection) in MDG (C) and CA1.SR (G) hippocampal subregions. CD31 capillary diameter in MDG (D) and CA1.SR (H). AQP4 positive capillary length in MDG (E) and CA1SR (I) was quantified in PND5 mice (24 h after *S. epidermidis* infection). AQP4/CD31 length ratios in MDG (F) and CA1SR. (L). Data are presented as median, 10–90th percentile. Statistical comparison between the *S. epidermidis* and saline groups was performed using the non-parametric Mann-Whitney test. ∗∗p < 0.01; ∗∗∗p < 0.001 (see also Figure S1).

To further delineate the involvement of LCN2 in hippocampal vessel alterations following infection, we performed a network analysis including the regulatory molecules previously identified by IPA. The IPA analysis clearly suggested a link between LCN2 and several brain processes and functions, such as endothelial dysfunction, extravasation, migration of endothelial cells and activation of astrocytes (Figure 3A). Therefore, we asked whether upregulation of LCN2 is associated with alteration of the cerebrovascular unit. We previously showed that *S. epidermidis* infection induced BBB dysfunction in the hippocampus^15^ and increased levels of LCN2 were reported to associate with increased BBB permeability in an animal model of subarachnoid hemorrhage.^20^ Thus, we next measured capillary diameter and length in CD31 stained sections (Figure 3B) to assess the impact of the bacterial infection on vascularization in the neonatal hippocampus. The capillary length of CD31 positive vessels was significantly increased 24h after *S. epidermidis* infection in the molecular layer of the dentate gyrus (MDG) (p = 0.004), with a trend in CA1 stratum radiatum (CA1SR) hippocampal subregion (p = 0.06, Figures 3C and 3G). Similarly, vessel diameter was increased in both MDG and CA1SR hippocampal subregions following infection (p = 0.002 and p = 0.0006 respectively, Figures 3D and 3H). As astrocyte endfeet are an active component of the BBB,^21^ serving as important regulators of vasoconstriction/dilation and cerebral blood flow,^22^ we also examined whether *S. epidermidis* has an impact on astrocyte endfeet capillary coverage by evaluating the length of AQP4 positive capillaries. The *S. epidermidis* infected mice demonstrated increased AQP4 positive capillary length in both MDG and CA1SR subregions (p = 0.01and p = 0.006, respectively, Figures 3E and 3I) and increased ratio of AQP4 to CD31 positive vessels in the CA1.SR (p = 0.004, Figure 3L), but not in the MDG (p = 0.9, Figure 3F). Systemic LCN2 levels were positively correlated with CD31 positive and AQP4 positive vessel length (Figure S1). Together, these results indicate that *S. epidermidis*, through a mechanism involving LCN2 localization in endothelial cells, alters the astrocyte endfeet coverage of capillaries in the hippocampus of neonatal mice, which might be linked to alteration of BBB permeability.

### Astrocyte reactivity following *S. epidermidis* infection

**Figure 4:**
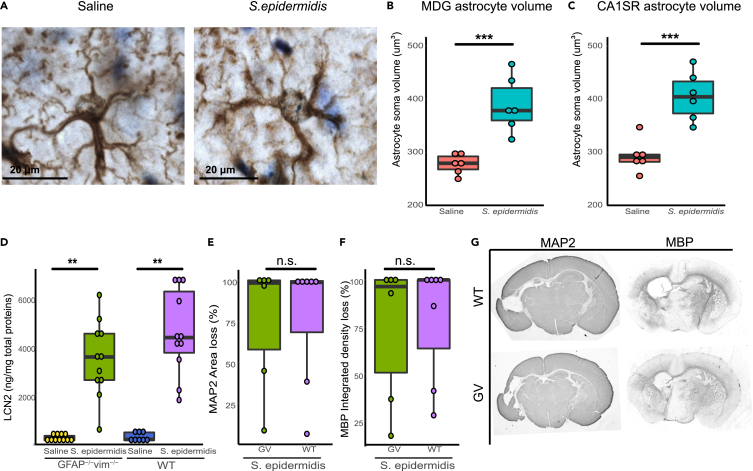
LCN2 upregulation following *S. epidermidis* infection in the neonatal hippocampus is independent of genetic attenuation of reactive gliosis (A–C) Representative images of astrocyte cell soma (100× oil immersion objective lens) in sections stained with GFAP of saline (left) and *S. epidermidis* (right) mice. GFAP positive astrocyte cell soma was measured in the MDG (B) and CA1SR (C) hippocampal subregion 24 h after *S. epidermidis* infection. (D–F) Hippocampal protein levels of lcn2 24 h after *S. epidermidis* infection (saline WT n = 8, *S. epidermidis* WT n = 11, saline *GFAP*^*−/−*^*Vim*^*−/−*^ n = 11, *S. epidermidis GFAP*^*−/−*^*Vim*^*−/−*^ n = 11). PND4 WT and *GFAP*^*−/−*^*Vim*^*−/−*^ pups were injected with *S. epidermidis* and subjected to hypoxia-ischemia 24h later. Hippocampal gray matter (E) and white matter (F) brain injury was assessed using microtubule-associated protein 2 (MAP-2) and myelin basic protein (MBP) staining respectively (*S. epidermidis* WT n = 7, *S. epidermidis GFAP*^*−/−*^*Vim*^*−/−*^ n = 6). (G) Representative images (4× objective lens) of PND14 *GFAP*^*−/−*^*Vim*^*−/−*^ and WT male mice brain sections stained with MAP-2 and MBP following *S. epidermidis* injection at PND4 in combination with hypoxia-ischemia 24h later. Data are presented as median, 10–90th percentile. Statistical comparison between *S. epidermidis* and saline for each group was performed using two-way ANOVA with Tukey’s multiple comparison post-hoc test or independent t-test. ∗∗p < 0.01; ∗∗∗p < 0.001 (see also Figure S2).

Alterations of astrocyte endfeet might reflect astrocyte activation^23^ and the IPA analysis indicated activation of astrocytes (Figure 3A). Hence, we also evaluated astrocyte reactivity in the hippocampus of saline and infected mice. Analysis of astrocyte volume in the hippocampus revealed a significant increase in the size of astrocyte soma in *S. epidermidis* infected mice compared to saline-treated animals in both MDG and CA1SR hippocampal subregions (p < 0.001 in both, Figures 4A–4C). As reactive astrocytes could be the source of LCN2,^24^ we next used mice with attenuated reactive gliosis because of lack of astrocyte intermediate filament (known also as nanoflament) proteins GFAP and vimentin (*GFAP*^*−/*^*Vim*^*−/−*^)^25^^,^^26^ to determine the link between LCN2 production and astrocyte reactivity. We found that LCN2 protein concentration was increased in both in WT and *GFAP*^*−/−*^*Vim*^*−/−*^ mice 24h following *S. epidermidis* infection (p = 0.001 in both, Figure 4D). We previously showed that *S. epidermidis* can increase the vulnerability to brain injury in neonatal mice^14^ and that the attenuation of reactive gliosis does not protect the neonatal brain following HI.^27^ However, the role of attenuated astrogliosis in *S. epidermidis*-sensitized brain injury is not known. We thus also asked whether brain injury was altered in *GFAP*^*−/−*^*Vim*^*−/−*^ mice following *S. epidermidis* infection combined with cerebral HI. Evaluation of changes in the white and gray matter following *S. epidermidis* infection combined with HI did not show a difference in injury in the hippocampus in *GFAP*^*−/−*^*Vim*^*−/−*^ compared to WT mice (Figures 4E–4G) or in *GFAP*^*−/−*^*Vim*^*−/−*^ compared to WT mice subjected to saline and HI (Figure S2). These results demonstrate activation of astrocytes in the hippocampus following *S. epidermidis* infection, however, reactive astrocytes did not seem to play a major role in the regulation of LCN2 protein expression in the brain or *S. epidermidis*-sensitized brain injury.

### Network analysis links CRP with blood proteomic changes in preterm infants

As inflammatory processes might be the driving force of neurodevelopmental conditions,^28^ identification of new markers and pathways associated with infection/inflammation would help to understand the pathophysiology mechanisms involved and to predict the neurological outcome.

Having identified LCN2 as a blood protein that is profoundly altered following *S. epidermidis* infection and highly associated with cerebrovascular changes in mice, we sought to determine the proteomic profile in blood of preterm infants experiencing an inflammatory response.

**Figure 5:**
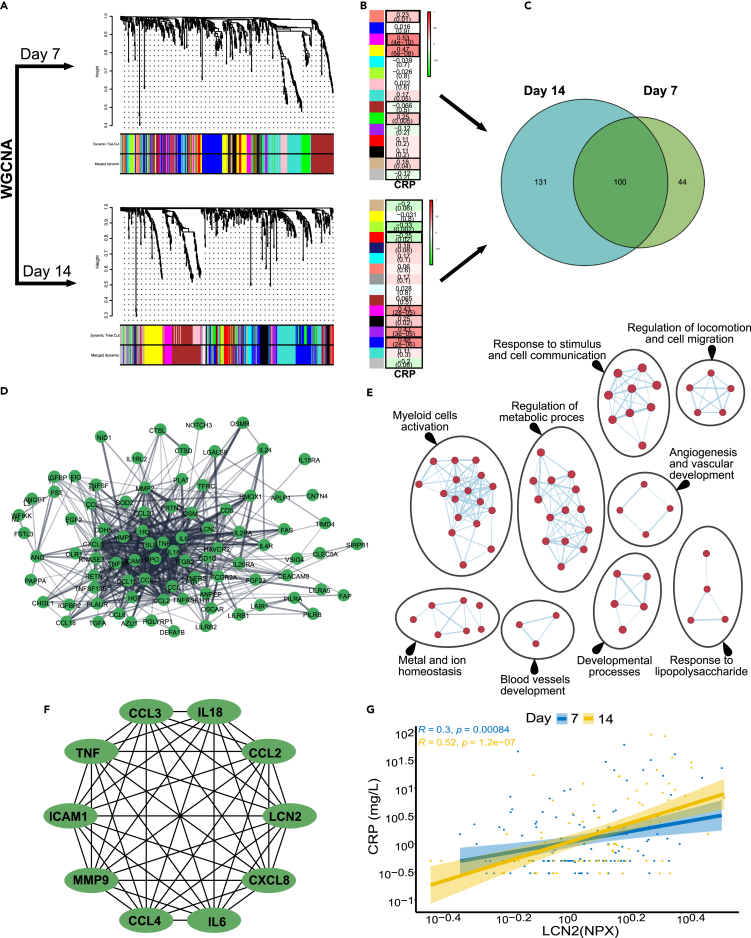
Preterm infant blood proteomic analysis using weight gene correlation network analysis (WGCNA) and identification of LCN2 (A–G) WGCNA identified 15 modules at day 7 (A, top panel) among which 5 modules were significantly correlated with CRP (B, top panel). At day 14, 16 modules were identified (A, bottom panel), among them 6 modules were significantly correlated with CRP (B, bottom panel). The numbers indicate the r score and in brackets the p value. (C) A total of 231 proteins identified at day 7 and 144 proteins identified at day 14 were associated with CRP. The intersection of the significant modules identified 100 proteins as common for both time points. (D) Protein-protein interaction (PPI) network constructed from the 100 overlapping proteins between the highly correlated proteins with CRP at day 7 and 14. (E) Enrichment map of significant gene ontology (GO) terms. Nodes represent gene sets. Highly similar protein sets are connected by edges, grouped in sub-clusters, and annotated automatically. (F) The top 10 high-degree proteins screened from the PPI network by using Maximal Clique Centrality (MCC) algorithm. The network was created using Cytohubba plug-in in Cytoscape. (G) LCN2 was positively correlated with CRP at both day 7 and day 14. See also Figure S3 and Tables S3 and S4.

Data on gestational week, sex and body weight in the human cohort are presented in Table S3. We analyzed blood proteomic data from 123 and 90 preterm infants at day 7 and 14 after birth respectively. CRP levels served as a measure of the inflammatory response. We performed two independent weighted gene co-expression network analyses (WGCNA) at 7 and 14 days after birth. WGCNA is a useful method to link co-expressed proteins modules to phenotypic traits (e.g., CRP).^29^ The full dataset of 123 infants at day 7 and 90 infants at day 14 was used for WGCNA analysis. At day 7, there were 15 modules identified (Figure 5A, top panel) five of which were significantly correlated with CRP (Figure 5B, top panel & Table S4). Similarly, at day 14, there were 16 modules identified (Figure 5A, bottom panel) and six of the modules were significantly correlated with CRP (Figure 5B, bottom panel & Table S4). The proteins identified by the WGCNA analysis were then compared to identify the common proteins that correlated with CRP at both time points (Figure 5C). The intersection of the significant modules identified 100 proteins that were common to both time points. The common proteins formed a large and interconnected network consisting of 95 nodes and 475 edges (Figure 5D). These proteins showed significant enrichment in multiple categories, such as response to stimulus and cell communication, myeloid cell activation, response to lipopolysaccharide, metal and ion homeostasis. Similar to the *S. epidermidis*-infected mice, the proteins that were highly correlated with CRP were enriched in pathways involved in vascular development and angiogenesis (Figure 5E). In addition, an interactome network analysis identified IL18, LCN2, CCL2, CXCL8, IL6, CCL4, MMP9, ICAM1, TNF and CCL3 as hub proteins in the network (Figure 5F). Intriguingly, LCN2 consistently and positively correlated with CRP at day 7 (R = 0.3, p = 0.00084) and day 14 (R = 0.52, <0.0001) (Figure 5G). There was no correlation between LCN2 and gestational week at birth (Figure S3A) or with CRP and gestational week at birth (Figure S3B). Thus, the results in preterm infants experiencing infection/inflammation, showing associations with LCN2 and vascular changes, are in line with our results in neonatal *S. epidermidis* infected mice.

### Circulating LCN2 levels correlate with inflammatory markers in both human and mice

**Figure 6:**
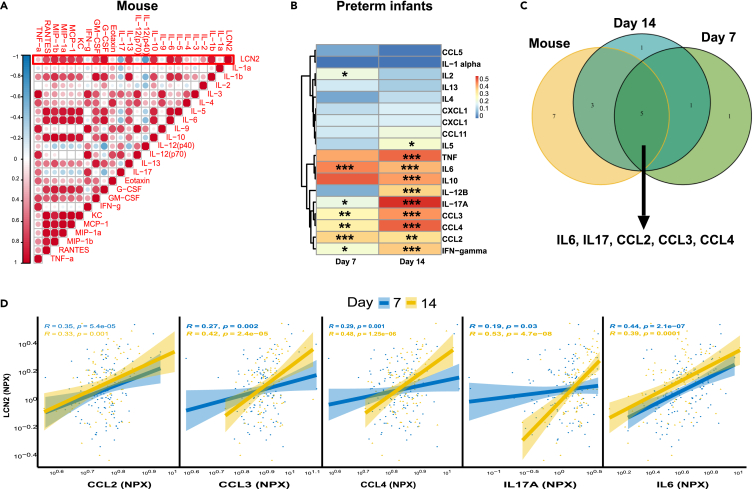
Circulatory Lipocalin 2, CRP and cytokine levels in mice and preterm infants (A) Correlation matrix of blood Lcn2 and chemokines and cytokine protein levels in PND5 mice 24 h after *S. epidermidis* infection (saline n = 9, *S. epidermidis* n = 10). (B) Spearman correlations between serum lcn2 and cytokines and chemokine in preterm infant blood. Spearman’s rank correlation coefficient is shown in the heatmap. P-value 0.05, 0.01 and 0.001 are indicated with ∗, ∗∗, ∗∗∗ respectively. (C) Venn diagram showing the overlap between cytokines significantly correlated with lcn2 in mouse and preterm infants on postnatal days 7 and 14. (D) Correlation of the common cytokines between human and mouse with lcn2 on postnatal days 7 and 14. Lcn2 data are presented as Normalized Protein expression (NPX). The correlation coefficient between two variables is presented in the graphs.

We previously showed that *S. epidermidis* induces upregulation of chemokines and cytokines in the blood of infected mice.^14^ In the current study we observed a positive correlation between LCN2 and Il-1β, IL-5, IL-6, IL-10, IL-13, G-CSF, GM-CSF, IFN-γ, CXCL1, CCL2, CCL3, CCL4, CCL5 and a negative correlation with IL-12 (p40) and IL-17 (Figure 6A), suggesting a link between LCN2 and systemic inflammatory responses following *S. epidermidis* infection. Next, we determined the correlation of infant serum levels of LCN2 with chemokines and cytokines at 7 and 14 days after birth (Figure 6B) and found that 7 days after birth, infant levels of LCN2 were significantly correlated with IL2, IL6, IL17A, CCL2, CCL3, CCL4, IFN-γ (Figure 6B). Similarly, 14 days after birth, LCN2 correlated with IL5, IL6, IL10, IL12B, IL17A, TNF, CCL2, CCL3, CCL4 and IFN-γ (Figure 6B). To evaluate if the cytokine/chemokine alterations following *S. epidermidis* infection in mice mirrored clinical inflammation in preterm infants, we overlapped the significantly LCN2-correlated cytokines from *S. epidermidis*-infected mice with the LCN2-correlated cytokines in preterm infants and found five cytokines (IL6, IL17, CCL2, CCL3, CCL4) to be common in mice and humans (Figure 6C). These five cytokines correlated with LCN2 levels in blood of preterm infants (Figure 6D).

### Differential protein expression link LCN2 with high CRP in blood of preterm infants

**Figure 7:**
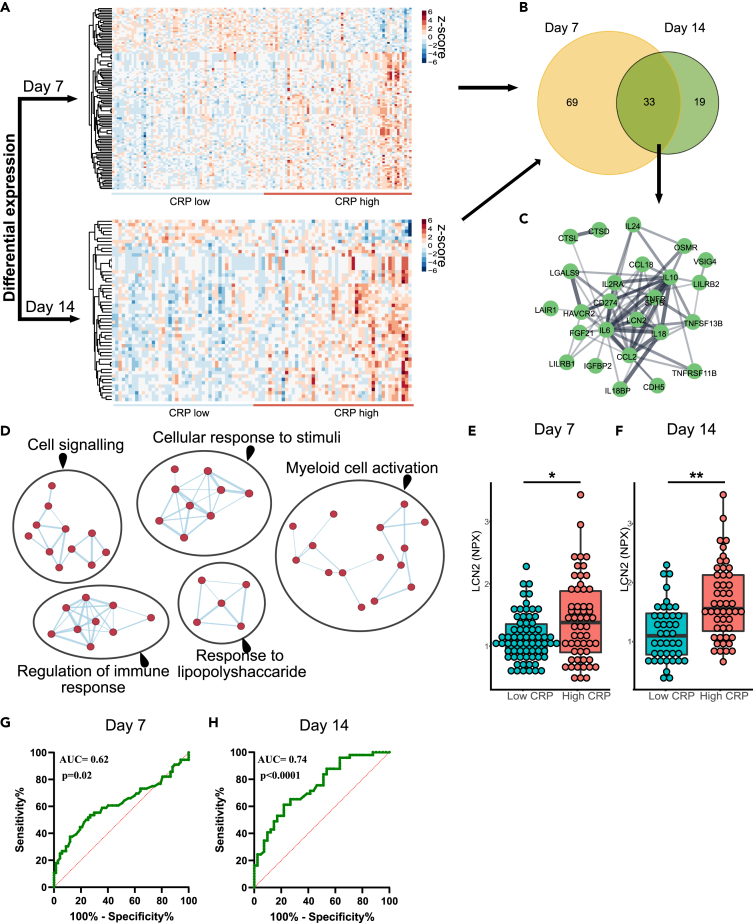
Differential protein expression and LCN2 identification in preterm infant blood (A) Heatmap of differentially expressed proteins at day 7 (A, top panel) and day 14 (A, bottom panel). (B) A total of 102 proteins were identified at day 7 and 52 proteins at day 14 to be differentially expressed between low and high CRP groups. The intersection of the significant proteins identified 33 proteins as proteins in common at both time points. (C) Protein-protein interaction network constructed from the 33 common proteins. The network was created using Cytoscape software. (D–H) Enrichment map of significantly gene ontology (GO) terms. Nodes represent gene sets. Highly similar protein sets are connected by edges, grouped in sub-clusters, and annotated automatically. LCN2 concentrations in blood of preterm infants at day 7 (n = 67 low CRP and n = 56 high CRP group) (E) and at day 14 (n = 42 low CRP and n = 49 high CRP group) (F). ROC curves of differentiating low CRP and high CRP group at day 7 (G) and day 14 (H). Data are presented as median, 10–90th percentile. Statistical comparison between *S. epidermidis* and saline for each group was performed using the non-parametric Mann-Whitney test. ∗p < 0.05; ∗∗p < 0.01. See also Table S5.

Next, we complemented the WGCNA analysis with investigation of differential protein expression. To investigate whether an increased CRP response was associated with LCN2 concentrations and blood proteomic changes in preterm infants, the median value of CRP (0.83mg/mL) was used to dichotomize preterm infants into low CRP (n = 67 and n = 42 at day 7 and 14, respectively) and high CRP (n = 56 and n = 49 at day 7 and 14, respectively) groups. At day 7 we identified 102 differentially expressed proteins (DEPs) and at day 14 we identified 52 DEPs (Figure 7A & Table S5). The intersection of DEPs at day 7 and 14 identified 33 proteins, including LCN2, in common (Figure 7B). The common proteins formed an interconnected network consisting of 33 nodes and 72 edges (Figure 7C). These proteins showed significant enrichment in numerous immune response processes and response to infectious agent (Figure 7D). As LCN2 was highly correlated with CRP and further identified by the DEP analysis, we compared LCN2 levels in preterm infants with low and high CRP at both time points. We found that high CRP levels were associated with upregulation of LCN2 levels at both day 7 (p = 0.019) and day 14 (p = 0.004) (Figures 7E and 7F). In addition, the LCN2 concentration discriminated infants with low CRP from those with high CRP at both day 7 (AUC = 0.62. 95% CI: 0.51 to 0.72, p = 0.02) and day 14 (AUC = 0.74. 95% CI: 0.64 to 0.84, p < 0.0001) (Figures 7G and 7H). Jointly, these results confirmed that LCN2 levels in the blood at 7 and 14 days after birth are increased in preterm infants with high CRP levels.

## Discussion

This study provides evidence for a role of LCN2 in neonatal infection/inflammation. Our results show that in infants born extremely preterm, the concentration of LCN2 in the systemic circulation is increased and in the first 2 weeks of life systemic LCN2 levels correlate with elevated levels of the inflammatory marker CRP. Both in infected mice and preterm infants, systemic LCN2 levels were associated with alterations in systemic cytokine and chemokine levels. Network and differential expression analysis further showed that proteins that were highly correlated with CRP were enriched in pathways involved in vascular development and angiogenesis. Similarly, in *S. epidermidis*-infected mice, LCN2 was increased in blood and the brain and IPA analysis revealed a link between LCN2 and cerebral vascular changes, such as endothelial dysfunction and migration of endothelial cells. Morphological analysis of brains showed increased length, increased diameter, and increased coverage by astrocytic endfeet of blood vessels in the brain of *S. epidermidis* infected mice and LCN2 was predominantly expressed in endothelial cells. Astrocytes were activated in *S. epidermidis*-infected mice but LCN2 protein expression in the brain was independent of astrocyte reactivity.

It is becoming clear that infection in the perinatal period has detrimental effects on brain development. Both epidemiological and experimental data pinpoint late-onset sepsis as a risk factor for adverse neurodevelopmental outcomes.^6^^,^^7^^,^^8^^,^^9^ Recently, we showed that a single LPS injection in neonatal mice resulted in increased repetitive stereotypic behavior and reduced sociability, suggesting a link between perinatal infection and development of autistic-like behaviors.^30^ Other studies have found that prenatal and postnatal exposure to infection increased the risk of schizophrenia and autism in both humans and animal models.^31^^,^^32^^,^^33^ Inflammatory mediators such as cytokines are often considered as common pathways associated with perinatal brain injury.^34^ In the current study, we found that IL17, IL6, CCL2, CCL3 and CCL4 were highly correlated with LCN2 in the blood of both *S. epidermidis*-infected mice and preterm infants. IL17 has been shown to exacerbate the inflammatory responses in sepsis, bronchopulmonary dysplasia, patent ductus arteriosus, necrotizing enterocolitis and brain injury.^35^ Of interest, IL17 increased in both the brain and meninges and was found to contribute to cognitive dysfunction in Alzheimer's disease.^36^ Further, IL17 expression was associated with BBB disruption.^37^^,^^38^ Similarly, CCL3 and CCL4 have been shown to contribute to demyelinating disease.^39^ CCL3 was elevated in preterm infants with necrotizing enterocolitis and in a mouse model.^40^ A positive correlation was found between CCL2 and LCN2 in an animal model of neuropathic pain.^41^ Of note, we found IL6, CCL2, CCL3 and CCL4 together with LCN2 to be hub proteins and highly linked with the CRP levels in preterm infants. Similarly, IL6 was upregulated in infants with high CRP levels.

Clinical signs of infection in neonates are often nonspecific, which can delay diagnosis and may increase the risk of morbidity and mortality in newborn infants.^17^^,^^42^ Measurement of serum CRP levels alone is unlikely to aid early diagnosis of late-onset infection^17^ and no currently used single infection marker or set of markers is sensitive and specific enough to identify neonates experiencing infection.^43^ CRP in combination with other markers of inflammation are used as indicators of infection in preterm infants.^43^ Specifically, the combination of IL6 and CRP has been suggested for identification of clinical sepsis in cases of negative bacterial culture.^44^ Preterm infants with high levels of CRP have higher levels of blood LCN2 and it has been suggested that LCN2 is a suitable marker for a variety of diseases in preterm infants, such as necrotizing enterocolitis,^45^ bronchopulmonary dysplasia^46^ and sepsis.^47^^,^^48^ Of interest, several past and ongoing clinical trials are testing LCN2 as a possible biomarker for septic patients (NCT02034240, NCT05374759, NCT02871544, NCT00711789). Although, the aim of the current study was not to identify biomarkers, the concomitant increase in LCN2, CRP and IL6, that we found in this study, might aid in recognizing preterm infants experiencing infection.

LCN2 is a member of the lipocalin family of proteins that bind iron-loaded siderophores that are required for bacterial growth.^49^ Under physiological conditions, the expression of *Lcn*2 mRNA in blood and brain is very low or absent and its expression occurs on injury and inflammation.^50^^,^^51^ In the adult brain, LCN2 protein was detected in several cell types such as neurons, microglia, astrocytes and vessels.^24^^,^^52^ In neonatal mice, we found LCN2 protein expression in brain vessels, but not in astrocytes. Although astrocytes become reactive following *S. epidermidis* infection, we provide evidence that activation of astrocytes does not affect LCN2 protein levels in the brain. By using *GFAP*^*−/−*^*Vim*^*−/−*^ mice, which exhibit attenuated reactive gliosis,^25^ we show that the increase in protein levels of LCN2 in the hippocampus after *S. epidermidis* infection are independent of this genetic attenuation of astrocyte reactivity and reactive gliosis. In contrast, our data suggest that increased levels of LCN2 in the brain may originate from brain vessels. However, in light of the increased BBB permeability following *S. epidermidis* infection^15^ and the fact that LCN2 is also produced by the liver^53^ and circulating neutrophils^54^ it is possible that peripheral sources also contribute to brain levels of LCN2.

Human and experimental studies have found local upregulation of LCN2 in several neurological conditions, such as ischemia/reperfusion-induced brain injury,^55^ intracerebral hemorrhage,^56^ traumatic brain injury^57^ and spinal cord injury.^58^ High levels of LCN2 were also found in the cerebrospinal fluid of patients with vascular dementia, a condition characterized by changes in memory and behavior as a consequence of pathological changes in the brain vasculature.^59^ Notably, we found that in *S. epidermidis*-infected mice high levels of LCN2 in blood were linked to disruption of the cerebrovascular unit. Consistently, network analysis of proteins highly correlated with CRP, including LCN2, in preterm infants suggested alteration of vascular development and angiogenesis. Of interest, it has been proposed that in ischemic brain damage, LCN2 secreted by endothelial cells contributes to neuronal cell death by interacting with its receptor and amplifying neuroinflammation and BBB disruption.^55^

Dysfunction of the gliovascular unit has serious consequences in several diseases, such as multiple sclerosis, epilepsy, stroke, vascular dementia, seizure, autism, and psychomotor retardation syndromes.^60^ The gliovascular unit maintains integrity of the BBB and regulates supply of cerebral blood flow.^61^ In several neurological conditions, inflammation plays a critical role in the disruption of BBB integrity as well as disease development and progression.^62^^,^^63^ High levels of LCN2 were suggested to cause BBB dysfunction.^52^ By investigating AQP4 as a marker of astrocyte endfeet, which are an active component of BBB, we show an increased astrocytic endfeet coverage of cerebral capillaries in the hippocampus following infection. In a rat model of brain edema, upregulation of AQP4 expression was observed together with BBB alteration^64^ and deletion of AQP4 reduced BBB leakage as well as inflammation.^65^ Thus, the increase in AQP4, combined with increased LCN2 expression, might be an underlying event associated with the BBB dysfunction in our mouse model of infection. However, although we found association between LCN2 and vascular disruption in neonatal mice, further studies are required to better delineate the pathophysiological involvement of LCN2 in the vascular changes and neurological disease.

Taken together, the results in this and our previous study^15^ point to LCN2 as a mediator of the interplay between brain blood vessels and glia cells that may underlie neuroinflammation and development of neurological conditions following *S. epidermidis* infection in neonates. The results highlight that LCN2 has the potential to become a complementary biomarker to aid the early diagnosis of infection in preterm infants. Because this is the first preclinical and clinical evaluation of LCN2 in this context, future investigation is required to better understand its role in the pathophysiological processes induced by *S. epidermidis* infection in the neonatal CNS.

### Limitations of the study

Although the experimental data suggest a strong association between LCN2 and cerebrovascular alteration, we cannot conclude a causal relation between LCN2 production and brain vascular changes. In addition, as confirmed infection was not retrieved in preterm babies, a limitation of our study is that LCN2 was related to infection/inflammation markers but not directly with infection and, thereby, we cannot conclude a clear correlation between LCN2 production and infection in preterm infants.

## STAR★Methods

### Key resources table

**Table:** 

REAGENT or RESOURCE	SOURCE	IDENTIFIER
**Antibodies**		

Anti- lipocalin-2 antibody produced in rat	Abcam	Ab70287; RRID:AB_2136473
Anti- cluster of differentiation 31 antibody produced in goat	R&D systems	AF3628; RRID:AB_2161028
Anti- glial fibrillary acidic protein antibody produced in rabbit	Dako	Z0334; RRID:AB_10013382
Anti- aquaporin 4 antibody produced in rabbit	BosterBio	BSBTPB9475; RRID:AB_2039734
Anti- microtubule-associated protein-2 antibody produced in mouse, clone HM-2	Sigma-Aldrich	M4403; RRID:AB_477193
Anti- myelin basic protein antibody produced in mouse, clone SMI-94	BioLegend	836504; RRID:AB_2616694
Alexa Fluor 488 donkey anti-rat IgG (H+L)	Life technologies	A21208; RRID:AB_2535794
Alexa Fluor 594 donkey anti-rabbit IgG (H+L)	Invitrogen	A21207; RRID:AB_141637
Alexa Fluor 647 donkey anti-goat IgG (H+L)	Invitrogen	A21447; RRID:AB_141844
Goat anti-rabbit IgG (H+L), biotinylated	Vector Laboratories	BA-1000; RRID:AB_2313606
Horse anti-goat IgG (H+L), biotinylated	Vector Laboratories	BA-9500; RRID:AB_2336123
Horse anti-mouse IgG (H+L), biotinylated	Vector Laboratories	BA-2001; RRID:AB_2336180
Peroxidase labelled goat anti-rabbit IgG (H+L)	Vector Laboratories	PI-1000; RRID:AB_2336198

**Bacterial and virus strains**		

*Staphylococcus epidermidis* 1457	Michael Otto’s laboratory^68^	

**Biological samples**		

Preterm infant blood samples	Randomized controlled trial MegaDonnaMega	ClinicalTrials.gov Identifier: NCT03201588)

**Chemicals, peptides, and recombinant proteins**		

Pentobarbital	APL	Cat#338327
Histofix	Histolab products AB	Cat#01000
Isopentane	Sigma Aldrich, Darmstadt, Germany	PHR1661
DAPI	Invitrogen	P36935
Antifade Mounting Medium	Vector Laboratories	Cat#00840-05
Target retrieval solution	Dako S1700, Glostrup, Denmark	S1699
3,3′-diaminobenzidine (DAB) solution	DAB EASY tablets, ACROS Organics™	AC328005000
Thionin solution	Sigma	T3387
Tryptan Blue	Sigma Aldrich,	Cat#302643
RLT Lysis buffer	Qiagen	Cat# 79216
miRNeasy Micro Kit	Qiagen	Cat# 217084
RNA library and transcriptome sequencing	Novogene Co., LTD (Cambridge, UK).	https://en.novogene.com/

**Critical commercial assays**		

ABC solution	VECTASTAIN Elite ABC HRP Kit	PK-6100
Pierce™ BCA Protein Assay Kit	Thermo Fischer Scientific	
Tandem Mass Tag (TMT-11plex) reagents	Thermo Fischer Scientific	
Dionex Ultimate 3000 UPLC system	Thermo Fischer Scientific	
QuantiTect Reverse Transcription Kit	Qiagen	
Fast SYBR master mix	Qiagen	
lcn2 primers	Qiagen	QT00113407
NGAL ELISA kit	Abcam	ab199083

**Deposited data**		

Dataset identifier PXD041926	ProteomeXchange, PRIDE database	**PubMed ID: 34723319**

**Experimental models: Organisms/strains**		

C57Bl/6J wild-type mice	Janvier Labs/ Charles River Laboratories	
GFAP-/- VIM -/-	University of Gothenburg Milos Pekny's laboratory	

**Software and algorithms**		

newCAST software	Visiopharm, Hørsholm, Denmark	https://visiopharm.com/visiopharm-digital-image-analysis-software-features/stereology/
Fiji software version 1.53c		
R software (version 1.4.1106)	https://www.r-project.org/	N/A
Perseus software	http://coxdocs.org/doku.php?id=perseus:start	
Cytoscape software (version 3.8.2)	Shannon, P., et al., 2003^69^	https://cytoscape.org/
DESeq2 v1.32.0	Love, M.I et al., 2014^70^	https://bioconductor.org/packages/release/bioc/html/DESeq2.html
WGCNA R package v1.68	Langfelder, P et al., 2014^31^	https://horvath.genetics.ucla.edu/html/CoexpressionNetwork/Rpackages/WGCNA/
Cytoscape StringApp	Doncheva, N.T et al., 2019^67^	https://apps.cytoscape.org/apps/stringapp
Enrichment Map	Merico, D et al. 2010^22^	https://apps.cytoscape.org/apps/enrichmentmap
Cyto-Hubba plug-in	Chin, C.H et al., 2014 ^71^	https://apps.cytoscape.org/apps/cytohubba
Proteome Discoverer version 2.4	Thermo Fisher Scientific	
Mascot search engine v. 2.5.1	Matrix Science, London, UK	
IPA	Qiagen	
GraphPad Prism 8	La Jolla, California, USA	

### Resource availability

#### Lead contact

Further information and requests for resources and reagents should be directed to and will be fulfilled by the corresponding author, Professor Carina Mallard (carina.mallard@neuro.gu.se)

#### Materials availability

This study did not generate new unique materials.

### Experimental model and study participant details

#### Animal model

In this study male C57Bl/6J wild-type (WT) mice, GFAP–/–Vim–/–^25^ and WT mice on a C57BL6−129Sv−129Ola mixed genetic background were used. To induce neonatal infection, mice were intraperitoneally injected with sterile saline or 3.5 × 10^7^ colony-forming units (CFU) of *S. epidermidis* at postnatal day (PND) 4. The age is equivalent to neonatal stage of development. All animal experiments conformed to relevant regulatory standards and were approved by the Gothenburg Animal Ethical Committee (No 663/2017).

#### Preterm infant samples

Preterm infant blood samples were collected within a randomized controlled trial MegaDonnaMega (ClinicalTrials.gov Identifier: NCT03201588). The Mega Donna Mega study protocol was approved by the Regional Ethics Review Board in Gothenburg (MEGADONNAMEGA 16-7) and the proteomic analysis was approved by the Swedish Ethical Review Authority (Dnr 303-11, T570-15). Written informed consent to participate was provided by the parents or legal guardians of all included infants. Sex and ages of human subjects are reported in the current manuscript (Table S3).

### Method details

#### Bacterial growth

To prepare the bacteria for injection, on the day of infection, an overnight culture was added to tryptic soy broth in a vented Erlenmeyer flask and shaken for approximately 4 h at 37°C and 240 rpm. The culture was centrifuged, and the resulting pellet was resuspended in endotoxin-free saline to reach 3.5 × 10^7^ CFU of *S. epidermidis* at specific CFU/ml concentrations.

#### Sample collection for histological evaluation

At PND5, C57Bl/6J pups were deeply anesthetized via intraperitoneal administration of pentobarbital (Pentacour) and perfused transcardially using saline. Brains were collected, fixed by immersion in Histofix (company) for one week, cryoprotected in 30% sucrose for 48 hours and frozen in cold isopentane (Sigma Aldrich, Darmstadt, Germany). Using a cryostat (Leica, CM 3050 S, Germany), 40-μm thick coronal sections were obtained. The first section of each series was randomly chosen by using a random table and the section sampling fraction (SSF) was ¼.

#### Immunofluorescence

To increase epitope retrieval, selected brain sections were placed in citrate buffer at 85°C for 40 min, blocked in 4% normal donkey serum and incubated in 0.2 % Triton X-100 with rat anti- lipocalin-2 (LCN2, 1:300; Ab70287; Abcam), goat anti- cluster of differentiation 31 (CD31, 1:300, AF3628, R&D systems) and rabbit anti- glial fibrillary acidic protein (GFAP, 1:250, Z0334, Dako ) or aquaporin 4 (AQP4, 1:200, BSBTPB9475, BosterBio) antibodies overnight at 4°C. Sections were then incubated in corresponding secondary antibodies produced in donkey and labelled with Alexa Fluor 488, 594 and 647 (1:200, Invitrogen) at room temperature for 2 h, counterstained with DAPI solution and mounted on slides using Antifade Mounting Medium (Vector Laboratories). Brain sections were examined and visualized using laser scanning confocal microscope Zeiss (LSM 800). Z-stacks of images were captured from brain sections with z-plane step size of 2.0 μm using 20× air objective lens and processed using Fiji software version 1.53c to create maximum projection of z-stacks for presenting the results. For a detailed view, a z-stack was captured with a 63x oil immersion objective using 2x zoom and z-plane step size of 0.2 μm. 3D visualization was created using Fiji software version 1.53c.

#### Immunohistochemistry

Three sets of sections from C57Bl/6J wild-type mice were stained for glial fibrillary acidic protein (GFAP), cluster of differentiation 31(CD31) and aquaporin 4 (AQP4) respectively. The sections were rinsed in PBS (2 × 10 min) and incubated in a target retrieval solution (Dako S1700, Glostrup, Denmark) for 40 min at 85 °C. Sections were then rinsed (2 × 10 min in PBS), endogenous peroxidase was blocked (3% H_2_O_2_, 10 min), and then rinsed again in washing buffer (0.25% triton in PBS for 20 min). After that, sections were incubated overnight with rabbit anti-GFAP, goat anit-CD31 and rabbit anit-AQP4 polyclonal antibodies as primary antibodies (diluted 1:500 in washing buffer). The next day, sections were rinsed with washing buffer (2 × 10 min) and incubated in solutions of corresponding secondary antibodies (1:200) for 2 h at room temperature. Subsequently, sections were rinsed in washing buffer (2 × 10 min) followed by incubation in ABC solution (VECTASTAIN Elite ABC HRP Kit, Peroxidase, Standard, PK-6100) for 1 h and rinsed in washing buffer for 20 min. In the last step, incubation in 3,3′-diaminobenzidine (DAB) solution (DAB EASY tablets, ACROS Organics™) for 1 min was performed. Finally, the sections were mounted on gelatin-coated slides, dried for 20 min, re-hydrated in demineralized water for 2 min, counterstained with 0.25% thionin solution (thionin, Sigma T3387), dehydrated through a graded series of alcohol (96%, 99%), cleared in xylene for 10 min and coverslipped.

#### Measurement of the size of astrocyte soma

Quantification of the size of astrocytes was performed on GFAP stained sections by measuring the volume of the cell soma with a 3D nucleator method in two subregions of the hippocampus, the CA1 stratum radiatum (CA1.SR) and the molecular layer of dentate gyrus (MDG). Delineation of CA1.SR and MDG subregions were performed according to the mouse brain atlas by a 5 × objective lens using the newCAST software (Visiopharm, Hørsholm, Denmark) modified for stereology with a digital camera (Leica DFC 295, Germany) and a motorized stage (Ludl Mac 5000, US). By using 3D nucleator, the number of half-lines was set at 6 and the mode was vertical uniform random (VUR) based on the assumption of rotational symmetry of astrocytes. Volumes of GFAP-immunopositive astrocytes were estimated using a 100 × oil-immersion objective lens. For each animal, 50-80 astrocytes were randomly sampled by using the optical dissector method.

#### Quantification of vascular parameters in the hippocampal CA1.SR and MDG subregions

The length density (Lv) of CD31^+^ and AQP4+ capillaries were determined by estimating the length of capillaries using a Global Spatial Sampling method. The estimated Lv of capillaries was done using a light microscope 63× oil- immersion objective lens. Briefly, in a three- dimensional sampling box, isotropic virtual planes with a fixed-plane separation distance (d = 20 μm) were systematically and randomly projected onto the area of interest. The box height was 20 μm with a top guard zone of 5 μm. A capillary was defined as a vessel with a diameter <10 μm. The Lv of CD31^+^ and AQP4+ capillaries were measured by the number of intersections between the virtual planes and the capillaries (Figure 4B). The Lv of the capillaries was calculated using the following formula:

$$L_{V}≔=\frac{2\cdot p\left(box\right)}{avg\;a\left(plane\right)}\cdot \frac{\sum Q}{\sum P_{ref}}$$

where Lv is the length density of the CD31^+^ or AQP4+ capillaries; ΣQ is the sum of intersections between the test lines and the capillaries; p (box) is the number of box corners (4); avg a (plane) is the average of the plane area; ΣP is the sum of the box corners hitting the area of interest.

The capillary diameter was measured in two hippocampal subregions (CA1.SR and MDG) using a 100× oil-immersion objective lens. This measurement was performed on 60-80 capillaries per region per animal which were sampled unbiased and randomly using an optical disector probe with a height of 25 μm. The selection criteria were the in-focused outer wall of the capillary and being entirely or partially inside the unbiased counting frame and not crossing the exclusion lines of the frame.

#### Hypoxia-ischemia (HI) and immunohistochemistry

Cerebral HI was induced in GFAP−/−Vim−/− and WT mice on a C57BL6−129Sv−129Ola mixed genetic background at PND5. Pups were anesthetized with isoflurane and the left common carotid artery was ligated. After surgery, mice were returned to their dams for 1 h, before being placed in a chamber with circulating humidified air (36 °C). In the chamber, mice were exposed to 10 min of air, followed by 60 min of hypoxia (10% O2 in 90% N2) followed by another 10 min of air. Following the hypoxic exposure, pups were returned to their dams until sacrifice. The brains were collected at PND14 and kept in 6% buffered formaldehyde (Histofix; Histolab) until dehydration and paraffin embedding. For the immunohistochemistry analysis, brains were sectioned at 7-μm coronal thickness on a microtome and stained against microtubule-associated protein-2 (MAP-2; clone HM-2, 1:1,000; Sigma-Aldrich catalog # M4403) or myelin basic protein (MBP; clone SMI-94, 1:1,000; BioLegend catalog # 836504).

#### Brain injury analysis

Gray matter injury was quantified on sections stained for MAP-2, which labels neurons and dendrites, and white matter was quantified on sections stained for MBP, which labels myelin. Images were captured on a light microscope (Olympus BX60) using a 4X objective lens. The region of interest (ROI) with MAP-2 or MBP positive immunoreactivity in the hemispheres ipsilateral and contralateral to the ligated artery were outlined and measured with ImageJ software (v1.52a, NIH, USA). Gray matter analysis was performed in the hippocampus (3 levels including the dorsal hippocampus).

Myelinated areas were determined as integrated density of MBP-positive staining (i.e., the product of area and mean gray value in the ROI). MBP staining was measured at 2 levels of the hippocampal fimbria. The percentage of MAP-2 and MBP-positive tissue loss were calculated at each level as follows: [(contralateral side – ipsilateral side)/contralateral side × 100%]. The mean of the percentage tissue loss for all levels was compared between animals.

#### Sample collection for Tandem Mass Tag spectrometry and PCR

Twenty-four hours after *S. epidermidis* or saline injection, C57Bl/6J pups were deeply anesthetized via intraperitoneal administration of pentobarbital (Pentacour) and perfused transcardially using saline. Left and right hippocampi were dissected. One hippocampus per animal was used for proteomics analysis using Tandem Mass Tag spectrometry and the other hippocampus was used for PCR for RNA expression analysis.

#### Sample preparation for proteomic analysis

Proteomic sample preparation and analysis were conducted by the Proteomic Core Facility at the University of Gothenburg. Samples were homogenized on a FastPrep®-24 instrument (MP Biomedicals, OH, USA) for 5 repeated 40-second cycles at 6.5 m/s in lysis buffer containing 2% sodium dodecyl sulphate (SDS), 50mM triethylammonium bicarbonate (TEAB). Lysed samples were centrifuged at 21 100 xg for 10 min and the supernatants were transferred to clean tubes. Protein concentrations were determined using Pierce™ BCA Protein Assay Kit (Thermo Fischer Scientific) and the Benchmark™ Plus microplate reader (Bio-Rad Laboratories, Hercules, CA, USA) with bovine serum albumin (BSA) solutions as standards. A representative reference sample was prepared, containing equal amounts from all individual samples prepared. Aliquots containing 50 μg of total protein from each sample and reference were incubated at 56°C for 30 min in the lysis buffer with DL-dithiothreitol (DTT) at 100 mM final concentration. The reduced samples were processed using the modified filter-aided sample preparation (FASP) method.^66^ In short, the reduced samples were diluted to 1:4 by 8M urea solution, transferred onto Microcon-30kDa Centrifugal Filter Units (catalogue no. MRCF0R030, Merck) and washed repeatedly with 8 M urea and once with digestion buffer (0.5% sodium deoxycholate (SDC) in 50 mM TEAB). Free cysteine residues were modified using 10 mM methyl methanethiosulfonate (MMTS) solution in digestion buffer for 20 min at room temperature and the filters were washed twice with 100 μl of digestion buffer. Pierce trypsin protease (MS Grade, Thermo Fisher Scientific) in digestion buffer was added at a ratio of 1:100 relative to total protein mass and the samples were incubated at 37°C overnight. An additional portion of trypsin was added and incubated for another 4 h.

The peptides were collected by centrifugation and isobaric labeling was performed using Tandem Mass Tag (TMT-11plex) reagents (Thermo Fischer Scientific) according to the manufacturer’s instructions. The labelled samples were combined into one pooled sample, concentrated using vacuum centrifugation, and SDC was removed by acidification with 10% TFA and subsequent centrifugation. The labelled pooled sample was treated with Pierce peptide desalting spin columns (Thermo Fischer Scientific) according to the manufacturer’s instructions.

The purified desalted sample was pre-fractionated into 40 primary fractions with basic reversed-phase chromatography (bRP-LC) using a Dionex Ultimate 3000 UPLC system (Thermo Fischer Scientific). Peptide separations were performed using a reversed-phase XBridge BEH C18 column (3.5 μm, 3.0x150 mm, Waters Corporation) and a linear gradient from 3% to 40% solvent B over 18 min followed by an increase to 100% B over 5 min and 100% B for 5 min at a flow of 400 μL/min. Solvent A was 10 mM ammonium formate buffer at pH 10.00 and solvent B was 90% acetonitrile, 10% 10 mM ammonium formate at pH 10.00. The fractions were concatenated into 20 fractions, dried and reconstituted in 3% acetonitrile, 0.2% formic acid.

#### nLC-MS/MS

The fractions were analyzed on an orbitrap Fusion™ Lumos™ Tribrid™ mass spectrometer interfaced with Easy-nLC1200 liquid chromatography system (Thermo Fisher Scientific). Peptides were trapped on an Acclaim Pepmap 100 C18 trap column (100 μm x 2 cm, particle size 5 μm, Thermo Fischer Scientific) and separated on an in-house packed analytical column (75 μm x 35 cm, particle size 3 μm, Reprosil-Pur C18, Dr. Maisch) using a gradient from 5% to 12% B over 5 min, 12% to 35% B over 77 min followed by an increase to 100% B for 3 min, and 100% B for 10 min at a flow of 300 nL/min. Solvent A was 0.2% formic acid and solvent B was 80% acetonitrile, 0.2% formic acid. MS scans were performed at 120 000 resolution, m/z range 375-1375. MS/MS analysis was performed in a data-dependent, with a top speed cycle of 3 s for the most intense doubly or multiply charged precursor ions. Precursor ions were isolated in the quadrupole with a 0.7 m/z isolation window, with dynamic exclusion set to 10 ppm and duration of 45 seconds. Isolated precursor ions were subjected to collision induced dissociation (CID) at 35 collision energy with a maximum injection time of 50 ms. Produced MS2 fragment ions were detected in the ion trap followed by multinotch (simultaneous) isolation of the top 10 most abundant fragment ions for further fragmentation (MS3) by higher-energy collision dissociation (HCD) at 65% and detection in the Orbitrap at 50 000 resolutions, m/z range 100-500.

#### Proteomic data analysis

The data files were merged for identification and relative quantification using Proteome Discoverer version 2.4 (Thermo Fisher Scientific). Swiss-Prot *Mus musculus* database was used for the database search, using the Mascot search engine v. 2.5.1 (Matrix Science, London, UK) with MS peptide tolerance of 5 ppm and fragment ion tolerance of 0.6 Da. Tryptic peptides were accepted with 0 missed cleavage and methionine oxidation was set as a variable modification. Cysteine methylthiolation and TMT on peptide N-termini and on lysine side chains were set as fixed modifications. Percolator was used for PSM validation with the strict FDR threshold of 1%. Quantification was performed in Proteome Discoverer 2.4. The TMT reporter ions were identified with 3 mmu mass tolerance in the MS3 HCD spectra and the TMT reporter S/N values for each sample were normalized within Proteome Discoverer 2.4 on the total peptide amount. Only the quantitative results for the unique peptide sequences with the minimum SPS match % of 65 and the average S/N above 10 were taken into account for the protein.

#### GO enrichment analysis

Gene ontology (GO) pathway enrichment analyses were performed using Cytoscape StringApp^67^ and visualized in R software. GO terms with P adjusted to < 0.05 were considered to be significantly enriched. To overcome gene-set redundancy and help in the interpretation of large gene lists, Enrichment Map was used to visualize GO biological processes.

#### Ingenuity pathway analysis

IPA (Qiagen) was used to predict pathway regulation, upstream analysis and regulators. Only proteins with p < 0.05 and a fold change (FC) > |1.1| were used for the analysis. P-values were exported from IPA based on Fischer’s exact test (right-tailed for upstream analysis). All prediction graphs were exported from IPA, except for the canonical pathways analyses that were generated in GraphPad Prism 8 (La Jolla, California, USA) based on the data exported from the IPA.

#### Real-time quantitative reverse transcriptase PCR

Total RNA was prepared from the hippocampus lysates in RNase-free PBS using RNeasy Mini Kit (Qiagen), following the manufacturer's instructions and measured using NanoDrop 2000 (Thermo Fisher Scientific). RNA was reversed transcribed into cDNA using QuantiTect Reverse Transcription Kit (Qiagen). All cDNA samples were diluted with nuclease-free water to a final volume of 30 μl. Quantitative real-time RT-qPCR was performed with a Touch real- time cycler (Bio-Rad, Hercules, CA, USA). Each 20 μl reaction contained 10 μl Fast SYBR master mix (Qiagen), 2 μl of 10x Primer set (Qiagen) for lcn2 - QT00113407, 6 μl of H2O and 2 μl of cDNA. The PCR temperature profile was 95 °C for 2 min followed by 40 cycles of amplification (95 °C for 10 s and 60 °C for 30 s). The values for each gene were normalized to the concentration of cDNA in each RT sample.

#### Enzyme- linked immunosorbent assay (ELISA)

LCN2 protein concentration from hippocampal lysates and plasma was analyzed by enzyme- linked immunosorbent assay (ELISA) kit and standard (NGAL ELISA kit, ab199083, Abcam) as per manufacturer's instructions.

#### Protein measurement in preterm infant blood

Preterm infant blood samples were collected within a randomized controlled trial MegaDonnaMega (ClinicalTrials.gov Identifier: NCT03201588).^68^ The trial included infants born at less than 280/7 weeks gestational age at three Swedish hospitals (Gothenburg, Lund, or Stockholm) between December 2016 and December 2019. Peripheral serum samples for targeted proteomics were collected on postnatal days 7 and 14. Protein quantification was performed using the proximity extension assays technology^69^ and relative protein levels are reported in Normalized Protein Expression (NPX) units on a log2 scale. Details regarding the proteome profiling covering 538 unique proteins can be found elsewhere.^70^ Concentrations of infant plasma C-reactive protein (CRP) were retrieved from clinical records.

Data on birth weight, gestational age and sex were prospectively recorded accordingly to the study protocol. The Mega Donna Mega study protocol was approved by the Regional Ethics Review Board in Gothenburg (MEGADONNAMEGA 16-7) and the proteomic analysis was approved by the Swedish Ethical Review Authority (Dnr 303-11, T570-15). Written informed consent to participate was provided by the parents or legal guardians of all included infants.

#### Weighted gene co-expression analysis (WGCNA)

Standard WGCNA procedure was followed to create signed gene co-expression networks from the WGCNA R package v1.68^29^. Gene cluster dendrogram was constructed with a power value = 4 at day 7 and a power value= 5 at day 14. A total of 552 proteins were imported for the WGCNA analysis. The modules identified by WGCNA analysis were further associated, using Spearman correlation, with CRP independently at day 7 and day 14. The results identified at day 7 and day 14 were overlapped using Venn diagrams in R software and visualized using in Cytoscape software. Cyto-Hubba plug-in of the software Cytoscape was used to screen for hub proteins in CRP-correlated protein network based on the Maximal Clique Centrality (MCC) algorithm.

### Quantification and statistical analysis

#### Statistical analyses

Statistical analyses of mouse and human proteomics data were performed using Perseus software and visualized in R software (version 1.4.1106). For astrocyte morphology, lcn2 mRNA, CD31 and AQP4 vascular Lv measurements in WT mice, Student’s t-test was performed in R software. In GFAP−/−Vim−/− mice, LCN2 data analyses were performed using two-way ANOVA with Turkey post-hoc test using the functions “aov” and “TukeyHSD”. To determine the relationship between CRP and LCN2 in preterm infants, the mean levels of the two proteins for each individual in samples collected during postnatal day 7 and 14 were calculated, values were then log-transformed and Pearson correlation was applied. Spearman correlation was used to correlate the levels of LCN2 with cytokine and chemokine levels in preterm infant samples. Data are presented as median, 10–90th percentile. P < 0.05 was considered statistically significant.

### Additional resources

The study includes blood samples from human subjects, which are part of a clinical trial: ClinicalTrials.gov Identifier: NCT03201588.

## Data Availability

• Proteomic data have been deposited at PRIDE database and is publicly available. Accession numbers are listed in the key resources table. • The data reported in this paper will be shared upon request to the lead corresponding author (carina.mallard@neuro.gu.se). • Accession code for data set is provided in the key resource table. • This paper does not report original code.

## Supplemental information

Document S1. Figures S1–S3 and Tables S2 and S3

Table S1. Differentially expressed proteins, related to Figure 1

Table S4. Correlations at day 7 and day 14 in different modules, related to Figure 5

Table S5. Differential protein expression in preterm infant blood, related to Figure 7

Data S1. NC3Rs-ARRIVE-guidelines-checklist
