# Supplementary material for: Proteomics identifies lipocalin-2 in neonatal inflammation associated with cerebrovascular alteration in mice and preterm infants
Source: iScience. 2023 Jun 27;26(7):107217. doi: 10.1016/j.isci.2023.107217 (PMC10366453; doi:10.1016/j.isci.2023.107217)
Supplement: Document S1. Figures S1–S3 and Tables S2 and S3 [file mmc1.pdf]

iScience, Volume *26*

Supplemental information

Proteomics identifies lipocalin-2 in neonatal inflammation associated with cerebrovascular alteration in mice and preterm infants

Giacomo Gravina, Maryam Ardalan, Tetyana Chumak, Anders K. Nilsson, Joakim C. Ek, Hanna Danielsson, Pernilla Svedin, Milos Pekny, Marcela Pekna, Karin Sävman, Ann Hellström, and Carina Mallard

Supplementary figures 1-3 and tables 2 and 3.

Figure S1. Correlation of variables following *S. epidermidis* infection, related to Fig 3.

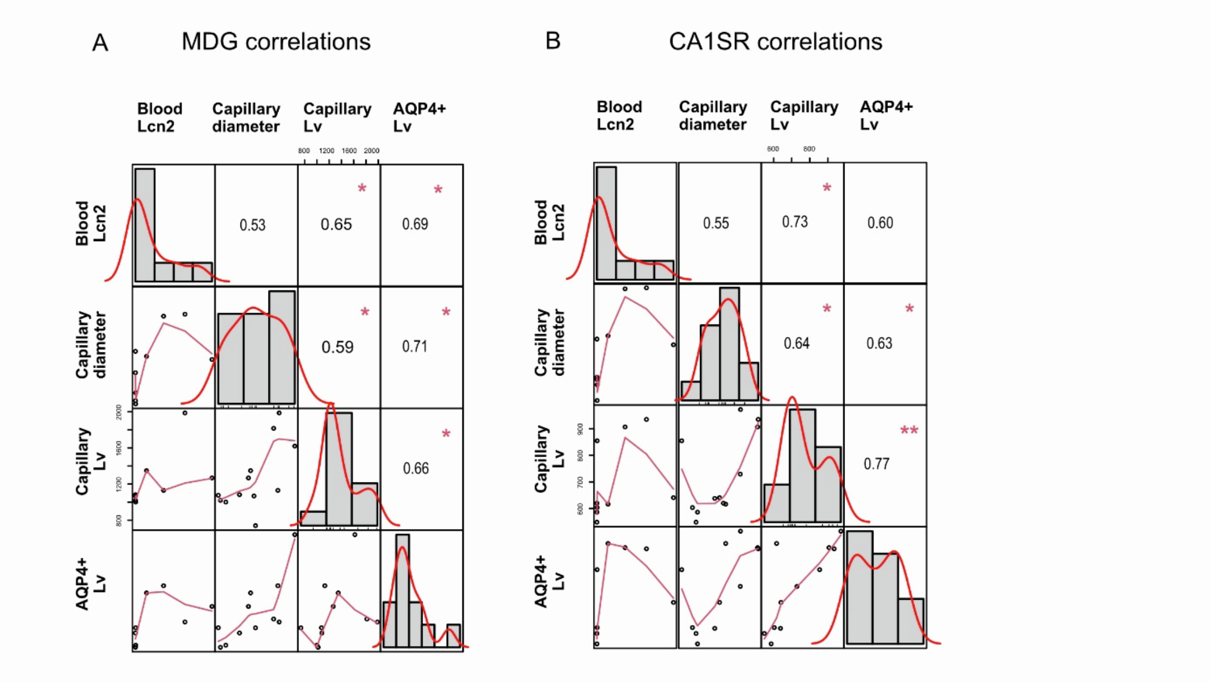

Figure S1. Correlation of variables following *S. epidermidis* infection. Correlation matrix between blood Lcn2, capillary diameter, capillary Lv. and AQP4+ Lv in MDG (A) and CA1SR (B). The distribution of the variables is shown on the diagonal. On the bottom of the diagonal, a bivariate scatters plot is shown whereas on the top of the diagonal, Spearman correlation values and significance are shown. V= volume, Lv= length density; p-value <0.05 and <0.01 are indicated with * and ** respectively

Figure S2. Baseline measurements of tissue loss in wild type and *GFAP*$^{-/-}$*Vim*$^{-/-}$ mice that underwent hypoxia-ischemia, related to Fig 4.

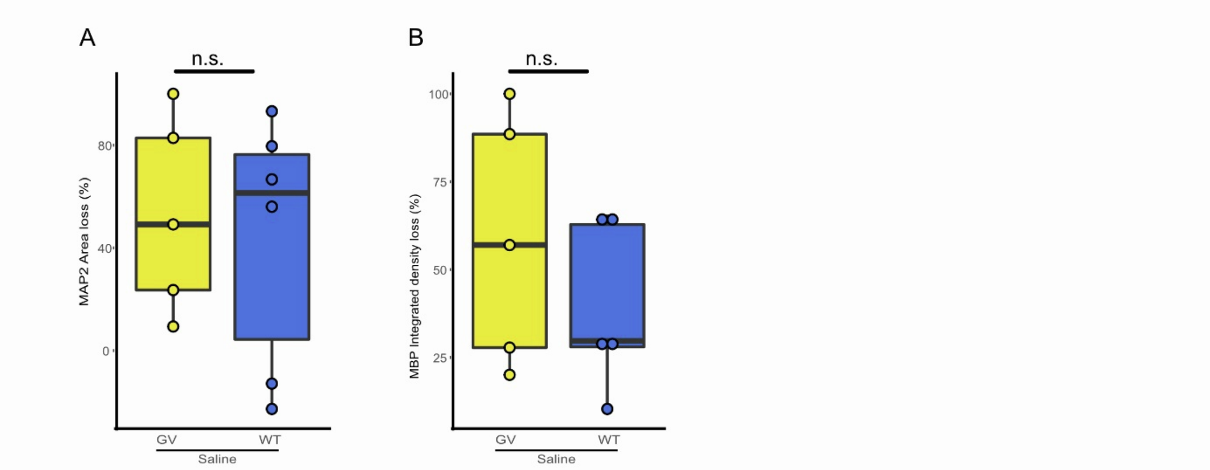

Figure S2. Baseline measurements of tissue loss in wild type and *GFAP*$^{-/-}$*Vim*$^{-/-}$ mice that underwent hypoxia-ischemia. PND4 WT and *GFAP*$^{-/-}$*Vim*$^{-/-}$ pups were injected with saline and subjected to hypoxia-ischemia 24 h later. Hippocampal gray matter (A) and white matter (B) brain injury was assessed by microtubule-associated protein 2 (MAP-2) (*Saline* WT n = 6, *saline GFAP*$^{-/-}$*Vim*$^{-/-}$ n= 5) and myelin basic protein (MBP) (*Saline* WT n = 5, *saline GFAP*$^{-/-}$*Vim*$^{-/-}$ n= 5). Data are presented as median, 10–90th percentile. Statistical comparison between wild type and *GFAP*$^{-/-}$*Vim*$^{-/-}$ mice for each group was performed using the non-parametric Mann-Whitney test.

Figure S3. Correlation between gestational week and LCN2 or CRP concentrations, related to Fig 5.

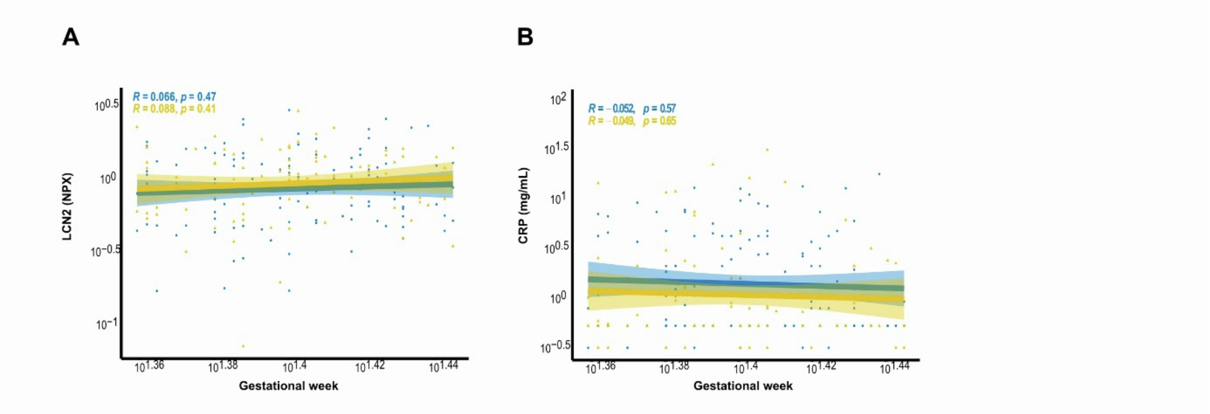

Figure S3. Correlation between gestational week and LCN2 or CRP concentrations. LCN2 (A) and CRP (B) do not correlate with Gestational weeks at either day 7 or day 14.

Table S2. Regulatory molecules, related to Fig 1.

Table S2. Regulatory molecules			
**Protein Name**	**Uniprot Description**	**Ratio SE/Ctrl**	**P-value**
**APOE**	APOE is an apolipoprotein, a protein associated with lipid particles, that mainly functions in lipoprotein-mediated lipid transport between organs via the plasma and interstitial fluids. APOE is a core component of plasma lipoproteins and is involved in their production, conversion and clearance. Apolipoproteins are amphipathic molecules that interact both with lipids of the lipoprotein particle core and the aqueous environment of the plasma. As such, APOE associates with chylomicrons, chylomicron remnants, very low-density lipoproteins (VLDL) and intermediate-density lipoproteins (IDL) but shows preferential binding to high-density lipoproteins (HDL). It also binds a wide range of cellular receptors including the LDL receptor/LDLR and the very low-density lipoprotein receptor/VLDLR that mediate the cellular uptake of the APOE-containing lipoprotein particles (By similarity).	0.2	0.93
**LCN2**	Iron-trafficking protein involved in multiple processes such as apoptosis, innate immunity and renal development (PubMed:12453413). Binds iron through association with 2,3-dihydroxybenzoic acid (2,3-DHBA), a siderophore that shares structural similarities with bacterial enterobactin, and delivers or removes iron from the cell, depending on the context. Iron-bound form (holo-24p3) is internalized following binding to the SLC22A17 (24p3R) receptor, leading to the release of iron and subsequent increase of intracellular iron concentration. In contrast, an association of the iron-free form (apo-24p3) with the SLC22A17 (24p3R) receptor is followed by association with an intracellular siderophore, iron chelation and iron transfer to the extracellular medium, thereby reducing intracellular iron concentration. Involved in apoptosis due to interleukin-3 (IL3) deprivation: iron-loaded form increases intracellular iron concentration without promoting apoptosis, while iron-free form decreases intracellular iron levels, inducing expression of the proapoptotic protein BCL2L11/BIM, resulting in apoptosis. Involved in innate immunity; limits bacterial proliferation by sequestering iron bound to microbial siderophores, such as enterobactin (PubMed:15531878, PubMed:16446425).	15.27	7.02E-06
**SERPINA3N**	The single human alpha1-antichymotrypsin gene (SERPINA3) is represented by a cluster of 14 individual murine paralogs.	2.09	0.0001
**ENG**	Vascular endothelium glycoprotein that plays an important role in the regulation of angiogenesis (PubMed:10625534). Required for normal structure and integrity of adult vasculature (By similarity).<br><br>Regulates the migration of vascular endothelial cells (PubMed:17540773). Required for normal extraembryonic angiogenesis and embryonic heart development (PubMed:10625534). May regulate endothelial cell shape changes in response to blood flow, which drives vascular remodeling and establishment of normal vascular morphology during angiogenesis (PubMed:28530658). May play a role in the binding of endothelial cells to integrins. Acts as a TGF-beta coreceptor and is involved in the TGF-beta/BMP signaling cascade that ultimately leads to the activation of SMAD transcription factors (PubMed:23300529). Required for GDF2/BMP9 signaling through SMAD1 in endothelial cells and modulates TGFB1 signaling through SMAD3 (By similarity).	0.73	0.006
**APOA1**	Participates in the reverse transport of cholesterol from tissues to the liver for excretion by promoting cholesterol efflux from tissues and by acting as a cofactor for the lecithin cholesterol acyltransferase (LCAT). As part of the SPAP complex, activates spermatozoa motility.	0.6	0.00003
**HMGCR**	Catalyzes the conversion of (3S)-hydroxy-3-methylglutaryl-CoA (HMG-CoA) to mevalonic acid, the rate-limiting step in the synthesis of cholesterol and other isoprenoids, thus plays a critical role in cellular cholesterol homeostasis.	0.89	0.02
**IGF1R**	Receptor tyrosine kinase which mediates actions of insulin-like growth factor 1 (IGF1). Binds IGF1 with high affinity and IGF2 and insulin (INS) with a lower affinity. The activated IGF1R is involved in cell growth and survival control. IGF1R is crucial for tumor transformation and survival of the malignant cell. Ligand binding activates the receptor kinase, leading to receptor autophosphorylation, and tyrosines phosphorylation of multiple substrates, that function as signaling adapter proteins including, the insulin-receptor substrates (IRS1/2), Shc and 14-3-3 proteins. Phosphorylation of IRSs proteins leads to the activation of two main signaling pathways: the PI3K-AKT/PKB	1.17	0.02

	pathway and the Ras-MAPK pathway. The result of activating the MAPK pathway is increased cellular proliferation, whereas activating the PI3K pathway inhibits apoptosis and stimulates protein synthesis. Phosphorylated IRS1 can activate the 85 kDa regulatory subunit of PI3K (PIK3R1), leading to activation of several downstream substrates, including protein AKT/PKB. AKT phosphorylation, in turn, enhances protein synthesis through mTOR activation and triggers the antiapoptotic effects of IGFIR through phosphorylation and inactivation of BAD. In parallel to PI3K-driven signaling, recruitment of Grb2/SOS by phosphorylated IRS1 or Shc leads to recruitment of Ras and activation of the ras-MAPK pathway. In addition to these two main signaling pathways IGF1R signals also through the Janus kinase/signal transducer and activator of transcription pathway (JAK/STAT). Phosphorylation of JAK proteins can lead to phosphorylation/activation of signal transducers and activators of transcription (STAT) proteins. In particular activation of STAT3, may be essential for the transforming activity of IGF1R. The JAK/STAT pathway activates gene transcription and may be responsible for the transforming activity. JNK kinases can also be activated by the IGF1R. IGF1 exerts inhibiting activities on JNK activation via phosphorylation and inhibition of MAP3K5/ASK1, which is able to directly associate with the IGF1R (By similarity). When present in a hybrid receptor with INSR, binds IGF1 (By similarity).		

Table S3. Clinical data on infants included in the study, related to Fig 5.

**Infants included in the randomized controlled trial MegaDonnaMega (ClinicalTrials.gov Identifier: NCT03201588)**		
	**Day 7**	**Day 14**
N. of preterm infants	123	90
Sex Male	71	55
Gestational week	25.25 weeks	25
Body weight	775.65 g	741
Treatment with AA:DHA	64	44
